# COVID-19 impact on the diagnosis of Inborn Errors of Metabolism: Data from a reference center in Brazil

**DOI:** 10.1590/1678-4685-GMB-2021-0253

**Published:** 2021-12-17

**Authors:** Fernanda Medeiros Sebastião, Kristiane Michelin-Tirelli, Fernanda Bender, Franciele Fátima Lopes, Inamara Moraes, Francyne Kubaski, Roberto Giugliani, Maira Burin

**Affiliations:** 1 Hospital de Clínicas de Porto Alegre (HCPA), Serviço de Genética Médica, Porto Alegre, RS, Brazil.; 2 Universidade Federal do Rio Grande do Sul, Departamento de Genética, Porto Alegre, RS, Brazil.

**Keywords:** COVID-19, Inborn Errors of Metabolism, lysosomal disorders, reference center, Brazil

## Abstract

The COVID-19 pandemic led to the reorganization of health care in several countries, including Brazil. Inborn Errors of Metabolism (IEM) are a group of rare and difficult to diagnose genetic diseases caused by pathogenic variants in genes that code for enzymes, cofactors, or structural proteins affecting different metabolic pathways. The aim of this study was to evaluate how COVID-19 affected the diagnosis of patients with IEM during the first year of the pandemic in Brazil comparing two distinct periods: from March 1^st^, 2019 to February 29^th^, 2020 (TIME A) and from March 1^st^, 2020 to February 28^th^, 2021 (TIME B), by the analysis of the number of tests and diagnoses performed in a Reference Center in South of Brazil. In the comparison TIME A with TIME B, we observe a reduction in the total number of tests performed (46%) and in the number of diagnoses (34%). In both periods analyzed, mucopolysaccharidoses (all subtypes combined) was the most frequent LD suspected and/or confirmed. Our data indicates a large reduction in the number of tests requested for the investigation of IEM and consequently a large reduction in the number of diagnoses caused by the COVID-19 pandemic leading to a significant underdiagnosis of IEM.

## Introduction

At the end of December 2019, in Wuhan, China, the whole world faced a new infectious disease, called COVID-19 caused by the SARS-CoV-2 virus. It would not take too long for the virus to spread worldwide, and on March 11^th^, 2020, the World Health Organization (WHO) classified it as a pandemic (World Health Organization, 2020).

In Brazil, the first COVID-19 case was reported on February 26, 2020 (Araujo et al., 2020; BRASIL, 2020a). Shortly thereafter a growing number of cases was recorded with almost 20 million people infected and over 540,000 deaths by mid-2021 (BRASIL, 2020b). In all of the states, four or more social distance measures were adopted, such as suspension of school classes and events, risk group quarantine, restriction of public transportation, and partial hold of the economy (Da Silva et al., 2020). As a consequence, a reorganization of the health care system was necessary, affecting the lives of a countless number of patients who use the public health system in Brazil. 

Inborn Errors of Metabolism (IEM), are a group of genetic diseases caused by pathogenic variants in genes that control enzymes, cofactors, or structural proteins affecting different metabolic pathways (Ismail et al., 2019; Elmonem et al., 2020). There are only a few reports relating COVID-19 and IEM, but it has been considered that some metabolic diseases such as amino acid disorders, organic acidemias, lysosomal and mitochondrial disorders are risk factors for the COVID-19 disease. We also know that IEM are rare and neglected diseases, difficult to diagnose, and require continuous clinical and laboratory monitoring. The lack of monitoring can worsen the disease course and the natural history (Lampe et al., 2020; Leoni et al., 2020; Mistry et al., 2020).

Since 1982, the Laboratory of Inborn Errors of Metabolism of the Medical Genetics Service (LIEM - MGS) of the Hospital de Clínicas de Porto Alegre (HCPA) has contributed to the biochemical understanding of many rare diseases and has become a reference center for the diagnosis of this group of diseases, receiving samples from all of Brazilian states and from other countries. The main specialty of LIEM-MGS is the diagnosis of Lysosomal Disorders (LDs), but the laboratory also frequently performs other tests for IEMs such as the diagnosis of classic galactosemia, type I tyrosinemia, biotinidase deficiency, congenital disorders of glycosylation, Smith-Lemli-Opitz Syndrome, among many other conditions.

The main aim of this study was to evaluate how COVID-19 affected the diagnosis of patients with IEM throughout the first year of the pandemic in Brazil.

## Material and Methods

### Samples

This is an observational study in which we compared the periods from March 1^st^, 2019 to February 29^th^, 2020 (TIME A) and from March 1^st^, 2020 to February 28^th^, 2021 (TIME B). Data such as the number of tests performed on urine samples (U), whole blood (WB), leukocytes (L), plasma (PL), dried blood spots (DBS), serum (S), fibroblasts (F) and the number of confirmed diagnoses per year performed by LIEM-MGS were also used in this analysis. We considered as a “confirmed diagnosis” those that are performed using the gold standard, usually performed via measurement of the enzymatic activity and/or specific biomarkers in leukocytes and/or fibroblasts, using fluorimetric or colorimetric assays, electrophoresis, chromatography, isoelectric focusing and other types assays/tests, and also cases with a positive molecular genetics diagnosis. Positive results in a single DBS sample were considered a diagnosis “to be confirmed”, and only considered as a “confirmed diagnosis” when analyzed in two different samples or combined with a positive molecular genetics results. The tests and diagnosis were divided into two different groups: Lysosomal Disorders (LDs) and other IEM (IEM-others).

### Statistical analyses

Mean and standard deviation were analyzed by SPSS version 22.0.

## Results

During TIME A the LIEM-MGS performed 7,422 specific tests for IEM. Of these, 6,750 (91%) were requested for IEM-LD investigation, the remaining 672 (9%) for IEM-OTHERS investigation. In TIME B the total number of tests performed for IEM investigation was 3,980. Of these, 3,497 (88%) are represented by IEM-LDs and the remaining 483 (12 %) were tests requested for IEM-others (Table 1). In the comparison of TIME A with TIME B, it was possible to observe a reduction of 46% in the total number of tests performed. Specifically for the investigation of IEM-LDs there was a reduction of 41% while for IEM-others there was a reduction of 28%.

**Table 1 -:** Tests performed by LIEM-MGS - Comparison of TIME A and TIME B.

	Enzyme / Metabolite	Disorder	Sample^*^	Time A	Time B	Percentages of change in the numbers of tests - TIME A compared to TIME B
**IEM-LDs**	α-Iduronidase	Mucopolysaccharidosis Type I / Mucolipidosis	L,F, L,DBS	109	37	<66%
	Iduronate sulfatase	Mucopolysaccharidosis Type II/ Multiple sulfatase deficiency	L,F, PL, DBS	235	84	<64%
	Heparan Sulfamidase	Mucopolysaccharidosis Type IIIA / Multiple sulfatase deficiency	L, F	78	14	<82%
	N-Acetyl-α-glucosaminidase	Mucopolysaccharidosis Type IIIB	L,F, PL,DBS	138	27	<80%
	Acetyl-CoA-α-glucosaminide-N-acetyltransferase	Mucopolysaccharidosis Type IIIC	L,F	80	13	<84%
	N-Acetylglucosamine-6-sulfatase	Mucopolysaccharidosis Type IIID / Multiple sulfatase deficiency	L,F	73	12	<84%
	N-Acetylgalactosamine-6-sulfatase	Mucopolysaccharidosis Type IVA / Multiple sulfatase deficiency	L,F,DBS	269	88	<67%
	β-Galactosidase	Mucopolysaccharidosis Type IVB / GM1 gangliosidosis / Galactosialidosis	L,F,DBS	545	283	<48%
	Arylsulfatase B	Mucopolysaccharidosis Type VI / Multiple sulfatase deficiency	L,F,DBS	353	136	<61%
	β-Glucuronidase	Mucopolysaccharidosis Type VII / Mucolipidosis	L,F, PL,DBS	286	129	<55%
	Galactocerebrosidase	Krabbe disease	L,F	95	56	<41%
	α-Mannosidase	α-Mannosidosis / Mucolipidosis	L,F, PL,DBS	61	38	<38%
	α-Fucosidase	Fucosidosis	L,F	2	2	=
	β-Mannosidase	β-Mannosidosis	L,F	2	4	**>50%**
	Palmitoyl protein thioesterase	Neuronal ceroid lipofuscinosis (CLN1)	L,F,DBS	509	192	<62%
	Tripeptidyl peptidase	Neuronal ceroid lipofuscinosis (CLN2)	L,F,DBS	511	200	<61%
	Hexosaminidases	GM2 gangliosidosis Tay Sachs / Sandhoff / Mucolipidosis	L,F, PL,DBS	180	118	<34%
	Hexosaminidase A MUGS	GM2 gangliosidosis B1 variant	L,F, PL,DBS	173	115	<33%
	Arylsulfatase A	Metachromatic leukodystrophy / Multiple sulfatase deficiency	L,F	85	39	<54%
	Lysosomal acid lipase	Lysosomal acid lipase deficiency / Wolman	L,F,DBS	44	37	<16%
	N-acetylgalactosaminidase	Schindler disease	L,F, PL	5	5	=
	α-Glucosidase	Pompe disease	L,F	67	39	<42%
	α-Galactosidase A	Fabry disease	L,F, PL,DBS	21	22	**>5%**
	β-Glucosidase	Gaucher disease	L,F,DBS	236	116	<51%
	Sphingomyelinase	Niemann-Pick A and B disease	L,F,DBS	80	51	<36%
	Neuraminidase	Sialidosis	F	2	1	<50%
	Chitotriosidase	Biomarker (Gaucher and others lysosomal disorders)	PL,DBS	656	529	<19%
	Urinary Glycosaminoglycans	Mucopolysaccharidosis	U	865	567	<34%
	Sialic acid	Sialidosis	U	34	12	<65%
	Oligosaccharides / Sialyloligosaccharides Chromatography	Oligosaccharidoses	U	358	225	<37%
	Glycosaminoglycans electophoresis	Mucopolysaccharidosis	U	521	254	<51%
	Sulfatide Chromatography	Metachromatic leukodystrophy / Multiple sulfatase deficiency	U	77	52	<32%
**IEM -others**	Succinylacetone	Type I Tyrosinemia	PL,U	199	146	<27%
	Orotic acid	Urea Cycle Disorders	U	54	30	<44%
	7-Dehydrocholesterol	Smith Lemli Optiz Syndrome	PL, S	30	19	<37%
	Carbohydrates chromatography	Galactosemia and others	U	45	31	<31%
	Transferrin Isoelectric Focusing	Congenital disorders of glycosylation (CDG)	S	209	131	<37%
	Galactose 1 phosphatase uridyl transferase	Classic Galactosemia	WB	51	39	<23%
	Biotinidase	Biotinidase deficiency	PL, DBS	70	78	**>11%**
	Sulfite test	Sulfite oxidase deficiency / Molybdenum cofactor deficiency	U	10	7	<30%
	ERLICH	Porphyria	U	4	2	<50%

^*^L: leukocytes; F: fibroblasts; PL: plasma; DBS: dried blood spots; U: urine; S: serum; WB: whole blood samples.

**Figure 1 -:**
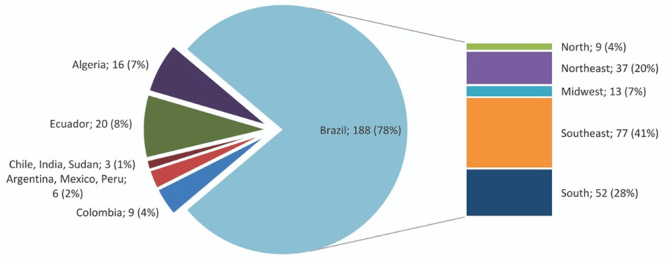
Patients distribution according to country and distribution of the Brazilian patients according to the Brazilian regions during TIME A.

In TIME A, the laboratory was able to diagnose 242 patients for 38 different types of IEM. Of these, 151 (62%) were confirmed and 91 (38%) required further confirmation in an additional sample. Of the 151 confirmed, 142 (94%) were IEM-LDs and 9 (6%) IEM-others. Of the 91 diagnoses that required further confirmation, 87 (96%) represent IEM-LDs and 4 (4%) IEM-others (Table 2). The mean age of the 242 patients (confirmed and or to be confirmed) ranged from 9.5 (±12) years for IEM-LDs and 6.3 (±11) years for IEM-others. 140 (58%) patients were male and 102 (42%) females.188 (78%) patients were from Brazil, followed by 20 patients from Ecuador (8%), 16 patients from Algeria (7%), 9 patients from Colombia (4%), 6 patients from Argentina, Mexico and Peru with 2 patients each (2%) and 3 patients from Chile, India and Sudan with 1 patient each (1%). The Brazilian patients were mainly from the Southeast (41%), South (28%), Northeast (20%), Midwest (7%), and North (4%) (Figure 1).

**Table 2 -:** Comparison of number of diagnoses corfirmed and to be confirmed performed by LIEM-MGS during TIME A and TIME B.

Disorder	Time A		Time B	
	Confirmed	To be confirmed	Confirmed	To be confirmed
α-Mannosidosis	1	0	0	0
Congenital disorders of glycosylation (CDG)	0	3	0	1
Biotinidase deficiency	2	0	2	0
Lysosomal acid lipase deficiency / Wolman	1	1	0	1
Fabry disease	4	0	2	0
Classic Galactosemia	2	0	1	1
GM1 gangliosidosis / Galactosialidosis / MPS IVB	0	7	0	5
GM1 gangliosidosis / Galactosialidosis	0	3	0	4
GM1 gangliosidosis	0	0	4	0
Galactosialidosis / MPS IVB	0	1	0	0
Galactosialidosis	1	0	0	0
Gaucher disease	18	2	10	2
Krabbe disease	8	1	5	0
Neuronal ceroid lipofuscinosis (CLN1)	4	0	1	0
Neuronal ceroid lipofuscinosis (CLN2)	5	6	10	14
Mucolipidosis I/II	0	8	0	2
Metachromatic leukodystrophy	7	0	3	0
Metachromatic leukodystrophy / Pseudo Arylsulfatase A deficiency	0	4	0	0
Metachromatic leukodystrophy / Multiple sulfatase deficiency	0	3	0	1
Mucopolysaccharidosis Types I,II,VII	0	3	0	1
Mucopolysaccharidosis Type I	8	2	3	1
Mucopolysaccharidosis Type II	27	9	8	1
Mucopolysaccharidosis Type IIIA	5	0	3	0
Mucopolysaccharidosis Type IIIB	7	10	3	0
Mucopolysaccharidosis Type IIIC	5	4	0	2
Mucopolysaccharidosis Type IIID	0	0	1	0
Mucopolysaccharidosis Type IVA	12	18	9	11
Mucopolysaccharidosis Type IVB	0	0	0	0
Mucopolysaccharidosis Type VI	11	2	17	4
Mucopolysaccharidosis Type VII	2	0	0	0
Niemann-Pick A disease	2	0	3	0
Niemann-Pick B disease	2	0	2	0
Niemann-Pick A and B disease	1	0	0	1
Pompe disease	6	0	0	1
Porphyria	0	1	0	0
Sandhoff	1	1	0	0
Sialidosis	1	0	0	0
Smith Lemli Optiz Syndrome	1	0	3	0
Tay-Sachs	0	1	4	2
Tay-Sachs B1	3	1	3	3
Type I Tyrosinemia	4	0	2	1
**Total**	**151**	**91**	**99**	**59**

Among the diagnoses of IEM-LDs, the mucopolysaccharidoses (MPS) represent 51% (77) of the confirmed diagnoses and 52% (45) of those requiring confirmation. Of the IEM-others, 44% (4) are from patients confirmed with type I tyrosinemia and 75% (3) patients with possible congenital disorders of glycosylation (to be confirmed).

**Figure 2 -:**
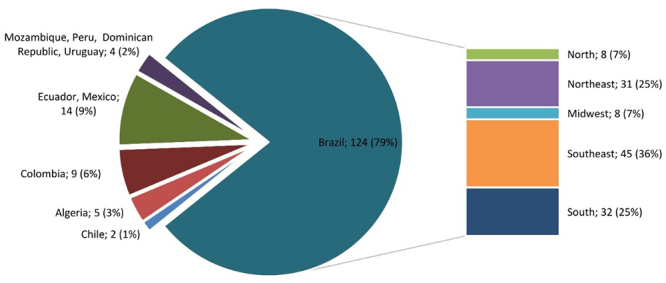
Patients distribution according to country and distribution of the Brazilian patients according to the Brazilian regions during TIME B.

In TIME B, 158 patients were diagnosed for 32 different types of IEM. Of these, 99 (63%) were confirmed and 59 (37%) required confirmation in an additional sample. Of the 99 confirmed patients, 91 (92%) are IEM-LDs and 8 (8%) are IEM-others. Of the 59 diagnoses that required confirmation, 56 (95%) represent IEM-LDs and 3 (5%) IEM-others (Table 2). The mean age of the 158 patients (confirmed and or to be confirmed) ranged from 8.5 (±12) years for IEM-LDs to 1.2 (±3.2) years for IEM-others. 81 (51 %) patients were male and 77 (49%) females. 124 patients were from Brazil (79%) followed by 14 patients from Ecuador and Mexico with 7 patients each (9%), 9 patients from Colombia (6%), 5 patients from Algeria (3%), 4 patients from Mozambique, Peru, Dominican Republic and Uruguay with 1 patient each (2%) and 2 patients from Chile (1%). The Brazilian patients were mainly from the Southeast (36%), South (25%), Northeast (25%), Midwest (7%) and North (7%) (Figure 2).

Among the diagnoses of IEM-LDs, MPS represented 48% (44) of the confirmed diagnoses and 36% (20) of those requiring confirmation. Of the IEM-others 37% (3) are from patients confirmed with Smith-Lemli-Opitz Syndrome.

Comparing both periods we can observe that the number of diagnoses was reduced by 34% (confirmed and/or to be confirmed) with a reduction of 36% in the confirmed cases and 34% of cases needing a confirmation. In the case of IEM-others the reduction in the number of diagnoses was of 11% in confirmed cases and 25% for cases that required confirmation. In both periods analyzed, MPS (all subtypes combined) represented the most frequent IEM-LDs confirmed and/or to be confirmed. For the confirmed diagnoses for IEM-others the most frequent disorders were type I tyrosinemia in TIME A and Smith-Lemli-Opitz Syndrome in TIME B.

The tests requested for the investigation of IEM-LDs had a total reduction of 48%. The most striking reduction was in the tests for the investigation of MPS type III (IIIC<84%, IIID<83%, IIIA: <82%; IIIB: <80%). The assay with a smaller reduction of request was the chitotriosidase enzyme assay (19%) and the assay for the analysis of the lysosomal acid lipase (16%). There was no reduction in the number of requests for the investigation of α-fucosidase and α-N-acetylgalactosaminidase (2 and 5 requests, respectively). There was an increase of 50% in the number of requests for the β-mannosidosis assay (from 2 to 4) and of 5% for α-galactosidase A assay (Table 2).

In the tests requested for the investigation of IEM-others, the total reduction was 28%. The greatest reductions were the Erlich test (<50%) and the assay of orotic acid (<44%). The assay for classic galactosemia, by the measurement of the activity of galactose-1-phosphate uridyl transferase had the smallest reduction (<23%) and the requests for the investigation of biotinidase increased by 11% (Table 2).

It is important to emphasize that in Brazil very few laboratories perform the investigation of IEM and that the LIEM is the reference center for these diagnoses. Figures 1 and 2 show that the LIEM covers all of the Brazilian regions and that it also covers some countries in South America, Asia and Africa. In regards to the diagnoses performed in samples from Brazil (confirmed and/or to be confirmed) there was a dramatic reduction of 34% in TIME B. Diagnoses from countries such as Algeria and Ecuador were also reduced (69% and 65%, respectively). Colombia kept the same number of diagnoses in both periods (Figures 1 and 2).

## Discussion

According to a survey carried out by EURORDIS through the Rare Barometer Programme, from April 18, 2020 to May 11, 2020 with 6,945 respondents in 36 countries in Europe with 1,250 different types of rare diseases, 83% of patients had some type of interruption in treatment and 6 out of 10 patients had no access to diagnoses (blood or imaging tests) during the pandemic (EURORDIS, 2020). In Brazil, the study by Schwartz (Schwartz et al., 2021) with 1,466 patients from all regions of the country, 98.8% of patients with different rare diseases had treatment interruption due to the cancellation of appointments, rehabilitation therapies, laboratory tests, transplants and surgeries.

In a study comparing a 3-months period between 2019 and 2020 in 16 diagnostic and treatment centers for IEM in 11 different countries with approximately 8,500 patients with IEM, it was shown that the laboratories had a reduction of 60% in the number of samples tested and of 80% in the number of new diagnoses. This reduction was even larger that the reduction we have seen in LIEM-MGS (14% and 46%, respectively) (Elmonen *et al*., 2020). The Italian study (Limongelli et al., 2021), reports on the difficulty of diagnosing in a network for rare disease which covers eight regions of Italy, showing a 32% reduction (1272 to 774 patients), this is also higher than the reduction seen at our center.

Unfortunately, the IEM were not the only underdiagnosed diseases in this period. In the Jiangsu Province, China, tuberculosis notifications from January to May 2020 were reduced by 36% when compared to the same period in 2019 (Liu et al., 2020). In the provinces of Daegu and Gyeongbuk, the epicenter of COVID-19 in South Korea, tuberculosis notifications decreased by 23% (Kwak et al., 2020). In Poland, the number of preliminary diagnoses of cancer in 2020 dropped by 31% compared to 2019 (Maluchnik et al., 2021). In the US, a cross-sectional study with more than 278,000 patients revealed that the number of cancer diagnoses was reduced by 46% during the COVID-19 pandemic (Kaufman et al., 2020).

Our group had already established the estimated frequency of LDs in Brazil in a study analyzing all diagnosis of IEM made at the LIEM from 1982 to 2015 in which 72% of these diagnoses were represented by IEM-LDs and 27% of these were mucopolysaccharidoses (Giugliani et al., 2017). Our current results show that IEM-LDs continues to be the most prevalent conditions representing 95% of the total confirmed and to confirm diagnoses during TIME A and 93% during TIME B, and that MPS still represent the most prevalent groups of diseases.

The drastic reduction in the number of requests for the investigation of MPS III can be explained by the usual diagnostic challenge, particularly in the early disease stages that can sometimes be confused with autism spectrum disorder, delaying the diagnosis of this disorder regardeless the COVID-19 pandemic (Wittkowski and Hare, 2017; Irigonhê et al., 2020). Other factors, as the establishment of alternative industry-sponsored diagnostic programs, may also be involved.

There was also a decrease in the number of requested tests for the diagnosis of IEM-LDs and IEM-others (48% and 28%, respectively). This smaller reduction in the number of tests requested for IEM-others as the drop in the average age from 6.3 to 1.2 years in patients with other types of IEM may be explained by the acute and more severe manifestations (generally neonatal or infantile) and also because of the Federal newborn screening program that screens for diseases such as galactosemia and biotinidase deficiency.

When we compare the diagnoses geographically, we do not observe a great variation in the distribution within the Brazilian regions. The reduction in the number of samples sent from other countries, such as Algeria (<69%) and Ecuador (<65%) can be explained probably due to the borders being closed during the pandemic. A questionnaire filled by physicians in the Saab study suggests that in countries such as Algeria, the abandonment of cancer treatment by some patients may be related to limitations in locomotion to treatment centers and the socio-economic impact of the COVID-19 pandemic in these families (Saab *et al*., 2020).

Our data indicate that in TIME B (COVID-19 period) there was a dramatic reduction in the number of test requests for the diagnosis of IEM and, consequently, in the number of diagnoses compared to TIME A (the period immediately before COVID-19). As there is no meaningful reason for a drop in the actual number of IEM cases, we conclude that during the COVID-19 pandemics there was a significant underdiagnosis of IEM. Health authorities should act to mitigate these negative impacts of the COVID-19 pandemic as IEM patients need to be promptly diagnosed and regularly monitored in order to benefit from the specific and general management measures available for these conditions.
